# Touch restriction during small-sided games in soccer—effects on physiological, physical and technical and tactical performance

**DOI:** 10.3389/fspor.2025.1705921

**Published:** 2025-10-14

**Authors:** Michael C. Rumpf, Johannes Jäger, Stefan Altmann, Matthias Lochmann

**Affiliations:** ^1^ Department of Sport Science and Sport, Friedrich-Alexander University Erlangen-Nürnberg, Erlangen, Germany; ^2^ Footballscience.net, Dreieich, Germany; ^3^ Sport Performance Research Institute New Zealand, AUT-University, Auckland, New Zealand; ^4^ Institute of Sports and Sports Science, Karlsruhe Institute of Technology, Karlsruhe, Germany; ^5^ TSG ResearchLab GmbH, Zuzenhausen, Germany

**Keywords:** one-touch play, football, training, soccer (football), coaching

## Abstract

Limited time and space availability during matches forces soccer players to utilize one-touch (1-touch) or two-touch (2-touch) passes to successfully maintain ball possession. Consequently, coaches replicate game demands during training, implementing touch restrictions into small-sided games (SSGs). 1-touch and 2-touch play increases the intensity and the perceived exertion of the SSGs in adult soccer. Physical variables that are associated with greater external load, such as sprinting or high-intensity running increased with touch restriction independent of skill level and player numbers. 1- and 2-touch play increase the players' technical engagement with the game (e.g., number of passes), however, resulting in positive (%successful passes) as well as negative (%unsuccessful passes) outcomes. Very scarce scientific resources exist with regards to tactical changes with touch restrictions in SSGs indicating indifferent results across age groups and limited tactical variables. Depending on the training goal, coaches can apply touch-restrictions to increase or decrease physical, physiological, technical and tactical activity of players.

## Introduction

One-touch (1-touch) play is an important component in soccer and it was suggested already in 1993 that it “is the winning requirement of football” (1). Indeed it was observed that individual world class players score in one-touch style in 44%–66% (2). Furthermore, between 60% and 80% (3–7) of all goals scored in international tournaments (3, 4, 7), and regular competition (5, 8) as well as domestic league play (6) were performed with 1-touch play. However, the importance of 1-touch play is not only seen in goal scoring. 1-touch passes allows attackers to play faster to circumvent defenders before shooting at the goal (9) and accounts for 37.5% up to 44.7% of all passes across positions and playing formations (10). In order to meet match demands, 1-touch play is utilized during small-sided games (SSG) as a popular coaching tool (11). A recent review article by de Assis Laria (12) published in 2024 provided insights into the physical and physiological effects on external load of players. The authors concluded that manipulating the number of touches allowed per ball possession influences players' physiological responses. More specifically, higher intensity is expected in SSG with fewer ball touches (12). On the other hand, physical responses are not influenced by this rule (12). Whilst acknowledging the provided information, the review considered published articles between 2010 and 2022, therefore missing recent scientific development in this topic and furthermore falls short on incorporating technical and tactical response with regards to touch restriction during SSG. This is a crucial aspect as touch restriction lead to tactical consequences (13) and 1-touch play was significantly different with various amount of opposing players applying pressure in real games (14). Furthermore, touch restriction was suggested to interfere with the technical performance during SSG and imposing a 1-touch limitation raised the number of involvements but also the frequencies of errors and balls lost (15). Consequently, touch restriction seemed to influence the technical and tactical performance of players. Therefore, the following paragraphs will elaborate in greater detail on the topic of touch restriction and its effect on physiological, physical, technical and tactical performance during SSG.

## Physiological variables

Touch restriction (1-touch and/or 2-touch-play) increases the intensity of the game in adult (16–22) football. That said, heart rate (HR) and blood variables associated with greater physiological strain are significantly greater in one and/or both the two restricted format (1- and 2-touch play) compared to free-play. More specifically, Dellal et al. (19) showed that %HRReserve was significantly greater in the 1-touch games compared to the free-play format, however only non-significantly greater compared to the 2-touch restriction. Players also perceive touch restrictions as more strenuous compared to the free play mode (19). Whilst not statistically investigated it seems that the increase in physiological variables [e.g., %HRMax (20, 21), %HRreserve (20, 21), blood lactate (20)] through touch restrictions is consistent across skill level and different players numbers (20) and number of bouts (21). More precisely, an upward trend is observed for blood lactate (20), %HRMax (20, 21), %HRReserve (20, 21) from free- play to 2-touch play and 1-touch play (20, 21). Furthermore, and supporting this notion, two scientific investigations (17, 18) were able to identify differences in the percentage of time spend in 91%–100%HRmax, which increased “likely” in the 2-touch-play format and was shown to be significantly different (17) from the free-play format (18). Lastly there was a “very likely” decrease in time spent <80%HRMax from 1st to 2nd half in the 2-touch-play compared to a “likely” decrease in the free play format (18). No clear trend with regards to the effect of touch restrictions on physiological variables were observed in two scientific investigations (16, 23) opposing scientific research is scarce. Only Roman-Quintana et al. (22) showed significantly greater mean heart rate, and percentage of mean heart rate in free-play format compared to the 2-touch games in amateur footballers playing 7 vs. 7 (22).

## Physical variables

The majority of research investigating the effect of touch restriction (1-touch and/or 2-touch-play) on physical variables indicate an increase in variables that are associated with greater external load in adult (17, 19–21, 24) and youth (25) football. Touch restriction increased sprinting (19, 21), high-intensity running (19), distance acceleration >4 m/s/s, however also percentage time walking (19, 22, 24, 26). Gimenez et al. (24) further showed significant difference in distance at low speed (>3.33–4.17 m/s) and time at moderate speeds (>4.17 < 5 m/s) comparing 1-touch vs. 2-touch vs. free-play. The lowest values were obtained in the 1-touch play followed by free-play and the 2-touch format. Whilst not statistically confirmed it seemed that the increase in physical variables (e.g., total distance, distance covered whilst sprinting, %total distance in sprint, %covered in high-intensity running) was consistent across skill level and different players numbers (20). Dellal et al. (20) showed an increase of all variables from the free-play format <2-touch-play <1-touch play. Similarly, Casamichana et al. (18) showed that work-to-rest ratio decreased in the free play mode “almost certainly” from the first to the second 6-minute game, whilst the identical variable was more stable (“unlikely” decrease) in the 2-touch-play mode (18). Furthermore, there was an “almost certain” increase in variables associated with lower external load (distance covered at 0.1–6.9 km/h), a “very likely” decrease in bands 7–12.9 and 13–17.9 km/h and a “likely” decrease in distance >18 km/h in the free-play format, whilst there was no clear trend in the 2-touch-play format indicating greater external load. There is a tendency regarding a lesser effect towards games with higher number of players (19, 27). Whilst not statistically analyzed (27), all physical variables (total distance, total distance covered in sprinting, % total distance in sprinting, total distance covered in high-intensity running, %total distance in HIR) were significantly different between the different kinds of touch restrictions (free play vs. 2-touch vs. 1-touch) in the 4 vs. 4 and 3 vs. 3, only the 1-touch rule resulted in a greater number of *p*-values of <0.001 across all variables compared to 2-touch and free-play (19). Interestingly, however, whilst non-statistically different, there seemed to be a trend over different bouts increasing total distance covered in high-intensity running with touch-restriction (free-play <2-touch <1-touch) and a decreasing trend for percentage of total distance covered in low and moderate intensity (free-play >2-touch >1-touch). Furthermore, 1-touch play was shown to inherent significantly higher total distance covered sprinting compared to 2-touch and free-play.

Research indicating opposing trend (i.e., greater physical strain in free-play) is limited. A decrease in total distance (26, 28), relative total distance (m/min) (28), jogging (3.6–14.3 km/h) (26), running (>14.4 km/h) (26), high acceleration (28), and increase in variables indicating lighter external loads such as walking (<3.6 km/h) (26) was observed with touch restriction in youth U9–U15 players (26). The latter variable however, seemed to display inconsistent responses as another scientific reference indicated greater distance walking in the free-play format (28).

## Technical variables

cTouch restriction during SSG influence the technical performance in adults (16, 19–21) as well as youth (25, 26, 28, 29) football. More specifically, touch restrictions increase the players' engagement with the game. Number of total passes (16, 25) increased with touch restriction, were highest with 1-touch rule (16), whilst simultaneously increasing the number of successful (26) as well as unsuccessful passes (16, 19, 28). The latter variable was also identified in different game formats involving different number of players (2 vs. 2; 3 vs. 3; 4 vs. 4) (19). Interestingly, only the 1-touch rule resulted in significantly different variables (%successful passes, number of lost balls, balls lost per minute play, total number of possessions, duels per minute of play, number of duels) from those obtained with the other two restrictions (2-touch and free-play) in all formats (19). Unsurprisingly, and as a result of the aforementioned variables [(un)-successful passes, as well as number of lost balls] the number of ball possession as well as the duration of possession change with touch restriction. Whilst the number of ball possession significantly increased, with decreasing touches (free-play <2-touch <1-touch) (21), the duration of possession showed opposing trend (free-play >2-touch) (29). Other variables such as number of duels and %lost balls were also shown to be significantly different from 2-touch and free-play (21). Bin Abdulla et al. (16) was also able to present shots on goal, number of crossings, interceptions and goals as significantly different in four conditions (1- vs. 2- vs. 3- vs. free-touch), however, no clear direction with regards to these variables was observed. The effect of touch restrictions seems to be independent of skill level and players numbers. However, this is not statistically confirmed, but a trend was observed for balls lost per min of play, total number of balls lost (free-play <2-touch <1-touch), %successful passes (free-play >2-touch >1-touch) with greater touch restriction (20).

There is little opposing research with regards to the effect of touch restriction on technical variables. Almeida et al. (29) investigated a 2-touch restriction in comparison to free play and presented ball touches, ball touches/duration, and ball touches/player involved as significantly higher in the free-play format. On the other hand passes/ball touches, passes/duration, and players involved/duration were significantly higher in the 2-touch games (29). Lastly, Coutinho et al. (26) reported a significant increase in the number of successful as well as unsuccessful passes only in the 1-touch format and depending on the age group. Other technical variables such as shots on target, goals scored were not different with touch restriction.

## Tactical variables

There are only scarce scientific resources with regards to the effect of touch restrictions and its effect on tactical performance. Only two investigations utilized youth (13, 26) soccer players and only limited conclusions can be drawn. Nevertheless, touch restrictions influenced the spatial exploration index (SEI), longitudinal and lateral synchronization (%) significantly in a 4 vs. 4+ goalkeeper (26). More specifically, the SEI was different in the U9, U11, and U17, the longitudinal synchronization in the U9, U13, U15, and the U17 and the lateral synchronization in the U15, U17, and U19. The authors concluded that coaches may use the 2-touch play in young age groups (U9–U13) as they seem less able to successfully cope with 1-touch restriction, while using 1-touch play in older age groups, due to their higher ability, to interact with environmental information (26). Sousa et al. (13), as the second scientific reference, compared free-play vs. a 2-touch restriction, showing that the restricted format reduced the offensive penetration significantly, however, increased width and length without ball as well as depth mobility. On the other hand, the defensive coverage was significantly lower, however concentration and defensive unity increased in the 2-touch format (13).

## Conclusion

Touch restrictions (1-touch and 2-touch) impacts players physiological, physical, technical and tactical performance. Coaches can control the intensity of the SSG through strategically implementing touch restrictions More specifically, coaches can start the main part of the training session without touch restriction (unlimited touches per player) followed by a two-touch restriction leading to higher heart rate and blood lactate responses. Touch restrictions will also increase the physical load during these games. Depending on the existing training day during two competitive matches (e.g., match day minus 1 or 3) coaches can regulate the physical load through controlling the number of touches during SSG. Again, if an increase in sprinting, running with a high pace as well as explosive movements such as high-acceleration is desired, coaches can implement touch restrictions. Coaches can increase the players’ technical engagement through implementing touch restrictions during SSG. This can be important in teams that inherit problems maintaining ball possession or coaching developing players (>14 years of age). Especially, a 2-touch restriction might favor a combination of increasing the technical involvement of individual player as well as maintaining a relative limited amount of pressure on the receiving player. However, coaches should also be cognizant that greater positive (%successful passes) as well as greater negative (%unsuccessful passes) outcomes will be experienced, which might have implications in training developing athletes with regards to psychological aspects. Coaches need to be aware of possible different effects of touch-restrictions across age groups, as it seems that younger players (U9–U13) are less able to successfully cope with 1-touch restriction compared to older players. Tactical responses to touch restriction were observed in maximizing playing space offensively, whilst decreasing defensive coverage. In sum, touch restrictions represent a simple yet powerful tool for coaches to manipulate intensity and focus during SSG, but their application should age-appropriate and carefully integrated into broader developmental goals.

## Limitation of the study

While this study provides valuable insights with regards to touch restrictions during SSG, some limitation should be acknowledged. Firstly, it needs to be mentioned that for different parts (e.g., physical, technical) the scientific information presented is a compilation of research in youth and adult footballers. Secondly, the playing level and experience might have influenced the physiological, physical, technical and tactical performance during the SSG. Combining different populations with different background seems to be common practice, especially in areas with limited scientific information available. However, the reader needs to be cognizant of that. Lastly, the information regarding the tactical variables derived from two scientific sources. Overall, further investigations should replicate the existing task-constraints (e.g., relative pitch sizes and goal configuration) to strengthen the effect of touch restrictions on players' physiological, physical, technical, and tactical parameters during SSG.
